# Current Advances in Radioactive Iodine-Refractory Differentiated Thyroid Cancer

**DOI:** 10.3390/curroncol31070286

**Published:** 2024-07-03

**Authors:** Fabio Volpe, Carmela Nappi, Emilia Zampella, Erica Di Donna, Simone Maurea, Alberto Cuocolo, Michele Klain

**Affiliations:** Department of Advanced Biomedical Sciences, University of Naples “Federico II”, 80138 Naples, Italy; fabio.volpe@unina.it (F.V.); emilia.zampella@gmail.com (E.Z.); ericadidonna5@gmail.com (E.D.D.); maurea@unina.it (S.M.); cuocolo@unina.it (A.C.); michele.klain@unina.it (M.K.)

**Keywords:** radioactive iodine, therapy, theragnostics, differentiated thyroid cancer, refractory DTC

## Abstract

Background: Differentiated thyroid cancer (DTC) patients have an outstanding overall long-term survival rate, and certain subsets of DTC patients have a very high likelihood of disease recurrence. Radioactive iodine (RAI) therapy is a cornerstone in DTC management, but cancer cells can eventually develop resistance to RAI. Radioactive iodine-refractory DTC (RAIR-DTC) is a condition defined by ATA 2015 guidelines when DTC cannot concentrate RAI ab initio or loses RAI uptake ability after the initial therapy. The RAIR condition implies that RAI cannot reveal new met-astatic foci, so RAIR-DTC metabolic imaging needs new tracers. ^18^F-FDG PET/CT has been widely used and has demonstrated prognostic value, but ^18^F-FDG DTC avidity may remain low. Fibroblast activation protein inhibitors (FA-Pi)s, prostatic-specific membrane antigen (PSMA), and somatostatin receptor (SSTR) tracers have been proposed as theragnostic agents in experimental settings and Arg-Gly-Asp (RGD) peptides in the diagnostic trial field. Multi-targeted tyrosine kinase inhibitors are relatively new drugs approved in RAIR-DTC therapy. Despite the promising targeted setting, they relate to frequent adverse-event onset. Sorafenib and trametinib have been included in re-differentiation protocols aimed at re-inducing RAI accumulation in DTC cells. Results appear promising, but not excellent. Conclusions: RAIR-DTC remains a challenging nosological entity. There are still controversies on RAIR-DTC definition and post-RAI therapy evaluation, with post-therapy whole-body scan (PT-WBS) the only validated criterion of response. The recent introduction of multiple diagnostic and therapeutic agents obliges physicians to pursue a multidisciplinary approach aiming to correct drug introduction and timing choice.

## 1. Introduction

While differentiated thyroid cancer (DTC) patients have an outstanding overall long-term survival rate, certain subsets of DTC patients have a very high likelihood of disease recurrence [1,2,3]. To evaluate the likelihood of recurrent or chronic illness in DTC patients, the American Thyroid Association (ATA) initial risk classification system has been proposed. Three risk categories (low, middle, and high) are assigned to patients. Furthermore, the ATA has suggested a dynamic risk categorization approach that considers imaging, biochemical, and clinical data gathered during follow-up. Radioiodine (RAI) diagnostic whole-body scanning (WBS) has been utilized in the past for DTC disease status assessment, but it has been replaced by a combination of neck ultrasonography (US) and serum thyroglobulin (Tg) measurement [1].

The current data demonstrate that patients with undetectable serum Tg levels have a high chance of achieving complete remission, and that a diagnostic workup may not be necessary in these cases [4,5]. Moreover, serum Tg, after some months of detectable levels, can tend to zero with no further actions.

An adequate uptake of RAI in the target tissue, defined as RAI avidity (RAI-A), is mandatory to obtain a successful RAI therapy. Low-risk disease and a post-surgical thyroid remnant are usually highly iodine-avid targets as they usually retain sodium–iodine symporter (NIS) expression.

Recently, the clinical application of RAI therapy has experienced a gradual decrease [6,7]. In particular, low-risk DTC should not be treated with post-surgery RAI ablation according to ATA guidelines [1]. Nevertheless, intermediate- and high-risk DTC may take advantage of RAI therapy administered for ablation purposes, or in cases of advanced disease, for metastasis therapy or palliation purposes [1].

The primary tumor size and the eventual lymph node metastases determine the administered radioiodine activity, but RAI-A is not guaranteed, especially in high-risk DTC or in the presence of known metastases [8,9,10].

Several factors have been associated with lower RAI-A of metastatic tissue, such as patient age, large tumor, histological type and high [^18^F]fluorodeoxyglucose uptake [11,12]. Furthermore, tumors exhibiting BRAF V600E or TERT promoter mutations are less likely to spawn iodine-avid metastases and are associated with poorer patient outcomes. The co-occurrence of these two mutational events in papillary thyroid cancer (PTC) has been found to be especially indicative of aggressive tumor features [8,13,14].

## 2. Defining Radioactive Iodine-Refractory Differentiated Thyroid Cancer

Despite the possibility of iodine uptake being altered ab initio in DTC [15], RAI therapy is still a cornerstone for the success of medium- and high-risk DTC treatment [1,2,16]. Iodine uptake may decrease with disease progression until further RAI administration becomes ineffective from a clinical point of view. In this condition, DTC can be considered refractory to RAI. RAIR-DTC is a relatively uncommon condition (four to five cases/million/year). RAIR-DTC is associated with a bad prognosis, and less than 10% of patients survive at 10 years (mean 3–5 years) [17].

Radioactive iodine-refractory DTC (RAIR-DTC) is defined by the ATA 2015 guidelines as a condition where DTC cannot concentrate radioactive iodine (RAI) at the time of initial treatment or loses its ability to concentrate RAI after initial therapy. RAIR-DTC also includes cases where only the local lesion concentrates RAI or there is disease progression and metastatic spread after high-dose treatment despite the ability to concentrate RAI [1].

While the refractory condition of DTC patients who lose the capability to concentrate RAI into the target lesion is well understood, more controversy surrounds cases where RAIR is associated with disease progression despite good RAI uptake. For these patients, evaluating the risk-to-reward ratio is crucial. After a cumulative dose of 600 mCi, the risk of side effects increases, while the likelihood of achieving a cure decreases. Therefore, the decision to continue RAI treatment should be made on a case-by-case basis, considering the patient’s previous response to RAI administration [18,19,20]. Table 1 summarizes all the conditions where the ATA 2015 guidelines define a DTC as RAIR.

Nevertheless, RAIR categories defined by ATA 2015 may appear over-restrictive in the view of a personalized medicine approach and should not be considered definitive. Martinique principles were proposed in 2019 when some experts proposed that the feasibility of RAI therapy in DTC patients should be discussed case by case, not excluding it a priori when a DTC patient falls in an ATA 2015 RAIR category. Indeed, RAIR definition criteria will be subject to evolution due to recent introduction of re-differentiation therapies [21].

The risk of RAIR-DTC can rise in elderly patients with an aggressive histological DTC subtype and with metastatic disease at the time of diagnosis. In these patients, cancer heterogeneity increases with RAI uptake inhomogeneity into target lesions, so RAI therapy can be less effective [3,22]. The prevalence of RAIR-DTC amounts to approximately 15% of DTC patients, particularly those with distant metastases at diagnosis and older age. BRAF and RAS kinase mutations are the more frequent alterations in follicular thyroid cancer (FTC) [8,14,23]. Extracellular signal-regulated kinase (ERK) or mammalian target of rapamycin (mTOR) activation pathways are the main mechanisms involved in RAIR with under-expression of sodium–iodine symporter (NIS) and the overexpression of glucose transporter 1 (GLUT1) [2,10,24,25]. From a functional point of view, DTC cells progressively lose the capability of accumulating iodine, but gain extra energetic substrates that can sustain the increased metabolic requirement of cancer cells.

## 3. Identifying RAIR

^131^I gamma emission can be used for diagnostic purposes with a whole-body scan (WBS) performed by a gamma camera. According to the ^131^I dose administered, WBS can be defined as diagnostic WBS (D-WBS) or post-therapy WBS (PT-WBS) according to low or high activity used. While PT-WBS can be considered a good negative detector of RAIR, the same consideration cannot be reserved to diagnostic ^131^I-whole-body scan (D-WBS). RAI activity, acquisition time, γ-camera model and TSH stimulation play a role in D-WBS accuracy and sensibility. In particular, low-administered-RAI activity for D-WBS acquisition would not allow the detection of all the RAI-avid foci [26,27,28].

Nevertheless, the added value of performing ^131^I-single-photon emission computed tomography (SPECT)–computed tomography (CT) has been debated since the introduction of this hybrid method. Some authors put the light on the better detection ability derived from the attenuation correction algorithms and from the morphological imaging acquired simultaneously [29,30,31]. In cases of equivocal findings on planar WBS images, PT-^131^I-SPECT/CT can differentiate remnant thyroid from lymph-nodal accumulation. Indeed, focal uptake of uncertain source may be defined as para-physiological or metastatic with fine body district localization [31,32,33]. Thus, the initial staging of DTC, as with patient risk assessment, can be corrected by additional imaging findings.

Patients who present negative imaging of ^131^I-WBS (despite D-WBS or PT-WBS) and abnormally elevated serum Tg levels should receive adequate attention, because this always indicates the presence of RAI-refractory disease.

RAIR often occurs in advanced DTC patients, when cancer heterogeneity increases along with aggressiveness. Additionally, RAI accumulation can vary lesion by lesion [34,35].

Molecular imaging reflects these changes accordingly. RAI accumulation decreases while ^18^F-fluorodeoxyglucose (FDG) uptake increases. This is called the “flip-flop” phenomenon and it is directly correlated with DTC dedifferentiation and aggressiveness [11,12,36,37].

^18^F-FDG positron emission tomography (PET)/computed tomography (TC) can be evaluated qualitatively by visual uptake detection and quantitatively by SUV estimation.

In RAIR-DTC, usually ^18^F-FDG uptake and SUV are increased compared to DTC. Nevertheless, SUV may be considered a good predictor of cancer growth speed in DTC [38,39].

^18^F-FDG DTC-positive findings are also correlated with poorer prognosis, as demonstrated by various scientific papers [2,12,36,39,40,41]. Some authors also suggest a good correlation between ^18^F-FDG uptake and the presence of BRAF v600e mutation in DTC cells [42,43].

## 4. Current Molecular Imaging and Care Options

Routine RAI imaging in clinical settings involves both regional and planar WBS and SPECT methods. However, there are no standardized quantitative methods for assessing response. Instead, response criteria often rely on visually assessed decreases in tumor uptake during post-treatment follow-up. It is important to consider the potential for functional tumor de-differentiation over the course of the disease when interpreting decreased RAI uptake in follow-up scans. To assess this possibility, ^18^F-FDG PET/CT scans, which reflect tumor glycolytic activity, should be used.

It is now possible to target aberrant cellular pathways and to provide additional treatment options for patients with otherwise poor prognoses due to the identification of multiple molecular alterations in advanced thyroid cancer.

For RAIR-DTC, the current standard of care involves treatment with tyrosine kinase inhibitors (TKIs).

The first-line setting includes both sorafenib and lenvatinib, as established by the National Comprehensive Cancer Network (NCCN) guidelines [44]. However, some patients manifest RET or NTRK fusions, and the standard of care has to be changed accordingly. More than half of patients show BRAF mutation, but the efficacy of BRAF inhibitors is not better than lenvatinib, and they are reserved to later therapy options [45].

Before initiating lenvatinib, blood pressure must be under control, but in cases of difficulty, sorafenib should be adopted. Selective RET inhibitors such as selpercatinib or larotrectinib should be preferred in patients with fusion detection. However, in cases of BRAF positivity, lenvatinib remains preferable, with BRAF inhibitors reserved for later lines of therapy. In the second-line setting, cabozantinib is also authorized and considered standard therapy.

The main challenges in managing RAI-refractory differentiated thyroid cancer (RAIR-DTC) include the onset of resistance and adverse events. To extend the efficacy of systemic therapy, local treatments such as surgery or external radiation should be considered for single progressing lesions. Although sorafenib and lenvatinib therapies are associated with adverse events, patients may achieve optimal outcomes and should be encouraged to adhere to treatment to avoid unnecessary dose reductions or treatment withdrawal.

## 5. Future Diagnostic and Therapeutic Perspectives

RAIR-DTC biochemical characteristics imply the need to research alternative targeted imaging tracers to iodine. Advanced cancer cells show some molecular pathway activation and mechanism similarities, so some tracers used in other cancer imaging could be adopted.

Integrin αvβ3 is involved in tumor angiogenesis and can be a potential imaging target for cancer growth using radiolabeled arginylglycylaspartic acid (RGD) peptides in DTC patients who had negative ^131^I-WBS, but elevated Tg levels [46,47,48,49]. Additionally, it has been suggested that ^99m^Tc-3PRGD2 uptake can predict the disease progression after initial RAI therapy in high-risk DTC patients [50]. RGD peptides can also be labeled with positron-emitting radionuclides for PET/TC application [51]. Chernaya et al. reported that BRAF mutation is linked with different expression levels of integrin receptors in DTC. In this scenario RGD imaging can be proposed under individualized conditions [52].

Prostate-specific membrane antigen (PSMA) ligands are a recent introduction in prostate cancer theragnostics [53,54,55]. PSMA overexpression has also been found in tumor neovasculature in various other tumors [56,57]. The expression of PSMA in thyroid tissue has been examined by some authors. Bychkov et al. enrolled 267 patients and found that PSMA was expressed in DTC neovasculature, but not in healthy tissue [58]. Similar results were found by Heitkotter and coworkers when comparing PSMA expression in thyroid cancer and benign thyroid diseases [59]. Hence, PSMA imaging in RAIR-DTC should be feasible. One study investigated PSMA uptake prospectively in 10 patients with 32 DTC metastatic lesions: 68Ga-PSMA PET/TC uptake was consistent (30/32 detected metastasis) and performance was superior to ^18^F-FDG PET/CT (23/32 detected metastasis) [60]. Verburg et al. in 2015 [61] and Lütje et al. in 2017 [62] demonstrated a possible role of ^68^Ga-HBED-CC-PSMA PET/CT for staging patients with RAIR-DTC metastases and for select patients eligible for PSMA radioactive labeled therapy. More recently, de Vries and coworkers explored the possible use of ^177^Lu-PSMA-617 therapy in five RAIR-DTC patients that showed ^68^Ga-PSMA PET/CT uptake in distant metastasis foci. Only two of them were considered eligible for ^177^Lu-PSMA-617 administration and only one of them established a temporary response [63]. These results need to be used to better define the possible role of PSMA ligands as a basis for future studies.

Somatostatin receptor (SSTR) types 2, 3, and 5 have been demonstrated in various studies in DTC cells and also in normal thyroid tissue and benign thyroid diseases [64,65,66,67,68]. Radiolabeled somatostatin analogues, such as octreotide and lanreotide marked with 68Ga-DOTA, have seen reasonably large use in PET/CT SSTR imaging in recent years, especially in neuroendocrine tumor (NET) imaging [69,70,71,72,73]. However, the role of SSTR tracers in RAIR-DTC remains unclear. In 2020, Donohoe and colleagues published a document on the appropriate use of the available nuclear medicine methods, including 68Ga-DOTATATE PET/CT and 177Lu-labeled SSTR tracers in RAIR-DTC. The committee stated that there was insufficient evidence to correlate Tg increase with 68Ga-DOTATATE PET/CT imaging positivity. Therefore 177Lu-labeled SSTR tracers should be considered in the therapeutic choices of RAIR-DTC patients that have demonstrated SSTR tracer imaging positivity [74].

Similarly to PSMA, radiolabeled choline PET/CT has found consistency in the diagnosis of prostate cancer. Thyroid uptake has been recorded in some ^18^F-choline PET/CT for prostate cancer diagnosis and staging [75,76]. ^18^F-choline PET/CT has also been investigated for detection of DTC metastases negative on ^18^F-FDG PET/CT. Piccardo et al. evaluated 25 patients with high-risk RAIR-DTC with both ^18^F-FDG and ^18^F-choline PET/CT. They found a good correlation with Tg doubling time and ^18^F-choline uptake. Thus, ^18^F-choline outperformed ^18^F-FDG in terms of sensitivity, specificity, and negative predictive value [77]. ^18^F-choline PET/CT should be considered in addition to ^18^F-FDG PET/CT DTC lesions.

More recently, attention has also moved to the tumor microenvironment (TME), a complex system composed of extracellular matrix, immune cells, fibroblast, endothelial cells, and signaling compounds. It has been demonstrated that the TME plays an important role in tumorigenesis and progression [78]. Of note, fibroblast function is shifted and promotes tumor growth, so these can be defined as cancer-associated fibroblasts (CAFs) and express the fibroblast activation protein (FAP) [78,79]. FAP can be targeted by FAP inhibitors (FAPis) and used in nuclear medicine theragnostic applications [80].

A possible RAIR-DTC application for FAPi has been explored by Chen and coworkers, who studied a population of 24 patients [81]. All of them underwent ^68^Ga-DOTA-FAPi-04 PET/CT and the detection rate was fairly good (87.5%). Ballal and co-workers compared ^68^Ga-DOTA-FAPi-04 PET/CT versus ^18^F-FDG PET/CT in 117 patients with RAIR-DTC and demonstrated superior performance in metastasis detection of radiolabeled FAPi over ^18^F-FDG [82]. After these results, Ballal et al. performed a pilot study aimed at evaluating a possible therapeutic use of ^177^Lu-DOTAGA.(SA.FAPi)2 in 15 RAIR-DTC patients that had failed on all of the standard options of systemic drugs [83]. At the end of the therapy cycles, the response rate was 92% and a complete response was achieved in 23% of patients.

^177^Lu-EB-FAPI was studied by Fu and coworkers in 12 patients with RAIR-DTC in a dose-escalation trial. The results, evaluated with RECIST 1.1 criteria [84], were a partial response in 25% of patients, stable disease in 58% of patients, and progression in 17% of patients [85].

Retinoic acids have been studied in thyroid function, and their impairment is often associated with iodine deficit and thyroid autoimmune disorders. Some authors suggested that retinoids are involved in gene regulation and NIS expression and potentially could be used in DTC treatment when RAI avidity decreases [86,87]. Pak and coworkers [88] and Groener et al. [89] explored the retinoic acid administration in RAIR-DTC patients for re-differentiation purposes and RAI administration eligibility. Both studies reported that a minority of patients responded to retinoid administration.

Selumetinib is an MAPK kinase (MEK) 1 and MEK2 inhibitor that has been proposed to reverse refractoriness to RAI. A cluster of RAIR-DTC patients were included in experimental selumetinib administration by Ho et al. [90]. Patient RAI uptake was studied by a ^124^I-PET/TC scan, performed before and after 4 weeks of selumetinib treatment, for dosimetry purposes. Eight (four with BRAF mutation and five with NRAS mutation) of the twenty patients received RAI due to the optimal RAI dose to lesions (≥2000 cGy). Five of eight obtained a partial response, while three achieved stability of disease [90].

Larson and coworkers also found an increase in RAI uptake after selumetinib administration in 20 RAIR-DTC patients studied with ^124^I-PET/TC scan [91].

The ASTRA phase III trial investigated selumetinib and RAI synergic administration in 233 high-risk DTC patients with high likelihood of RAIR. In sum, 78 patients received placebo and 155 patients received selumetinib and RAI adjuvant therapy. The tandem drug administration failed to improve the complete response rate in this patient cluster [92].

Sorafenib and lenvatinib are multi-targeted tyrosine kinase inhibitors (mTKIs) recently approved for use in RAIR-DTC [93,94,95,96]. Progression-free survival (PFS) achieved using these drugs is good, but neither overall survival (OS) nor quality of life (QOL) would match the patient’s needs. Numerous adverse events have been reported and the treatment is usually prolonged until progression, so the development of resistance has to be expected [94,95]. There is expanding evidence that mTKIs can induce a sort of re-differentiation in RAIR-DTC cells, promoting NIS exposition on cell membranes and re-inducing a possible RAI sensibility. Iravani et al. studied a re-differentiation protocol in six RAIR-DTC patients harboring the BRAF v600e mutation. The therapy was targeted to MEK with trametinib and the v600e mutation of BRAF with dabrafenib and trametinib. RAI uptake was demonstrated in four of six patients, and one of them achieved a complete response after therapeutic RAI administration [97]. Leboulleux and coworkers developed a phase II prospective trial based on re-differentiation therapy with dabrafenib and trametinib, followed by a fixed RAI administration of 5550 MBq. The RAIR-DTC status was demonstrated by a D-WBS prior to mTKI administration [98]. Eleven patients were enrolled and ten of them received RAI therapy. After 6 months, RECIST criteria defined a partial response in 20% of patients and stable disease in 70% of patients. Unfortunately, 10% of patients showed a progression of the disease. Metabolic assessment was performed with ^18^F-FDG PET/CT and results were similar to RECIST evaluation (partial response in 25%, stable disease in 63%, and progression in 13% of patients) [98]. Balakirouchenane et coworkers studied 22 patients undergoing re-differentiation therapy followed by RAI administration. They found a linkage between lower mTKi plasma concentration and RAI uptake [99]. Leboulleux et al. studied 24 patients with RAIR-DTC (confirmed by D-WBS) with small metastases that underwent a re-differentiation protocol with dabrafenib–trametinib tandem administration for 42 days [95]. A 5550 MBq RAI therapy was administered at day 28 after rh-TSH stimulation and a first evaluation of response was assessed by RECIST criteria after 6 months. If a partial response was reached, a second RAI could be administered after 6 or 12 months. Progression was diagnosed in 10% of patients, while partial or stable disease was achieved in 38% and 52% of patients, respectively. Ten patients received a second RAI administration: one of them obtained a complete response and six obtained a partial response at 6-month evaluation. One patient died because of progressive disease within 24 months. Despite the evidence of adverse events being common (96% of patients), the re-differentiation protocol was considered a good option for RAIR-DTC patients with small metastases.

## 6. A Case of Re-Differentiation

**Figure 1:**
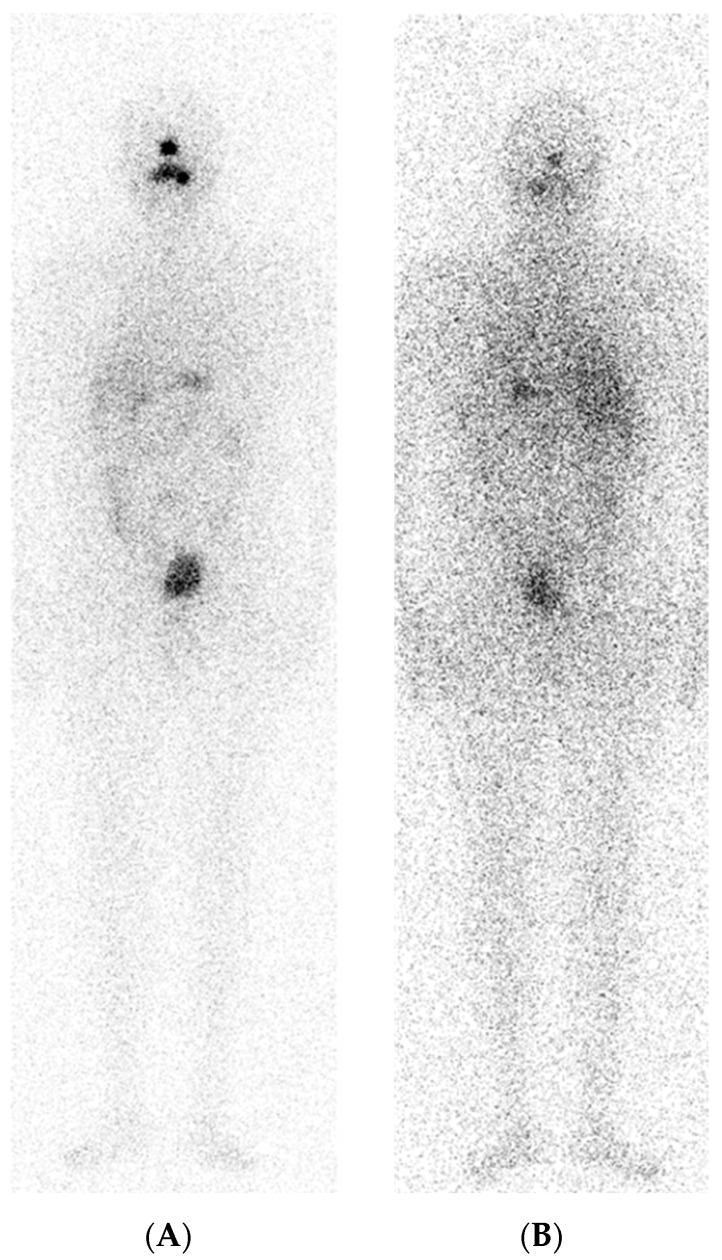
No evidence of pathological RAI uptake foci. (**A**) PT-WBS anterior view; (**B**) PT-WBS posterior view.

A 59-year-old man underwent total thyroidectomy in 2016 and a subsequent left cervical lymphadenectomy based on evidence of papillary thyroid carcinoma with lymph node metastases (pT1b N1b Mx). 5550 MBq of RAI were administered within 6 months from surgery. Nevertheless, Tg levels returned, detectable after some years from the first RAI therapy, so a second dose of 5550 MBq if ^131^I was administered. The PT-WBS did not show abnormal uptake foci (Figure 1), while Tg blood level was 378 pg/dL after FT4 withdrawal TSH stimulation and there was evidence of pulmonary nodules on CT examination.

The patient was defined as RAIR and the presence of BRAF v600e mutation was identified by molecular investigation. A re-differentiation protocol was attempted with the administration of dabrafenib and trametinib for 42 days. A third dose of 5550 MBq RAI was administered at the 28th day of dabrafenib and trametinib administration, under rhTSH stimulation.

**Figure 2:**
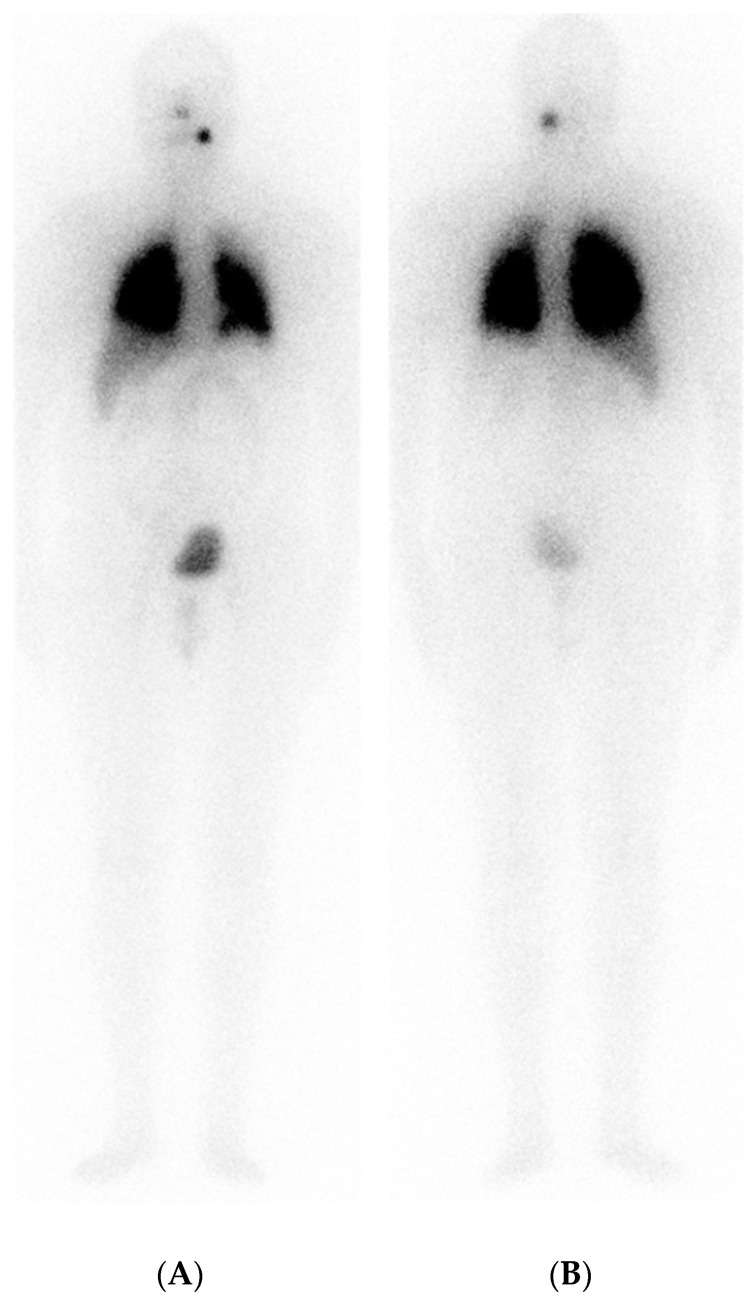
Focal RAI accumulation in upper-left cervical region: diffuse and intense RAI uptake in pulmonary field. (**A**) PT-WBS anterior view; (**B**) PT-WBS posterior view.

A PT-WBS scan demonstrated high RAI uptake in the pulmonary area and left cervical region (Figure 2).

**Figure 3:**
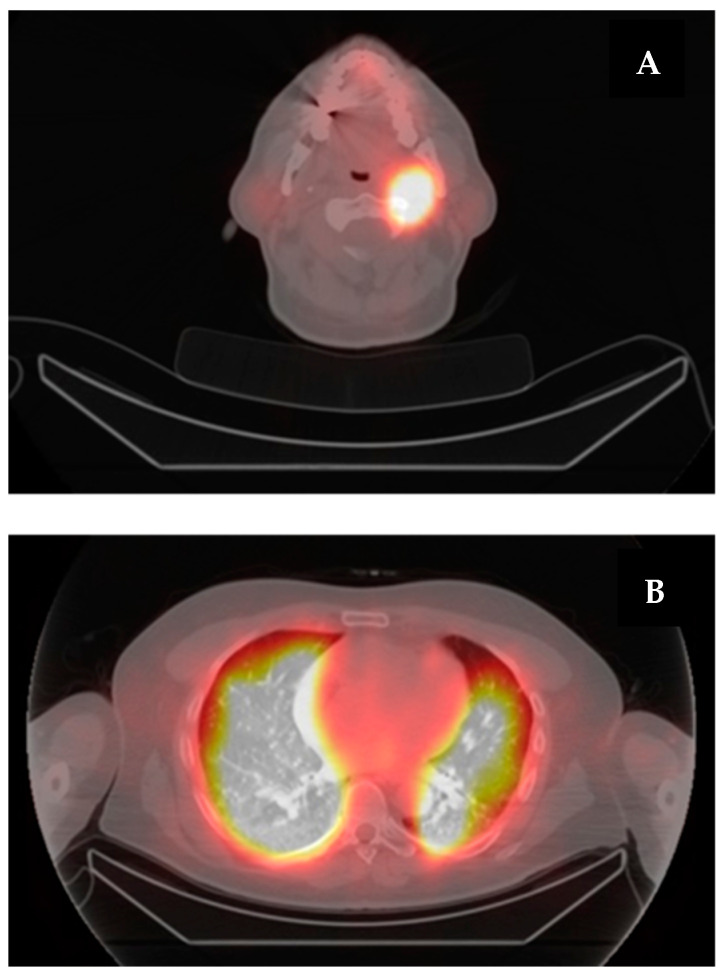
(**A**) High RAI accumulation in posterior mandibular lymph node. (**B**) Diffuse, intense, and bilateral RAI uptake in pulmonary tissue.

PT-SPECT/TC demonstrated RAI diffuse uptake in pulmonary parenchyma and left posterior mandibular lymph node (Figure 3). Tg blood levels also increased to 3183 pg/dL after rhTSH stimulation, suggesting that the re-differentiation protocol must have worked at different molecular levels.

## 7. Advanced RAIR-DTC Treatment

When cancer progresses, it accumulates mutations and acquires multiple drug resistance. In this setting, different molecular targets have to be explored to obtain a clinical benefit. In RAIR-DTC, MAPK pathway alterations are involved in cancer de-differentiation and proliferation, so several drugs have been tested in this condition [100].

Lenvatinib is a broad-spectrum TKI directed at vascular endothelial growth factor receptors (VEGFRs), fibroblast growth factor receptor (FGFR) 1–4, C-KIT, RET protooncogene, and platelet-derived growth factor receptor α (PDGFR-α). It was first approved for advanced hepatocellular carcinoma, but recently it has been introduced in RAIR-DTC therapy options. While overall response rate and disease control are acceptable, the main lenvatinib shortcoming is the onset of important adverse events that can interfere with therapy continuation [101].

Similarly to lenvatinib, sorafenib is an oral antiangiogenetic agent. In the DECISION trial, sorafenib resulted in a significant improvement in progression-free survival over placebo in a setting of RAIR-DTC patients who showed progression after RAI therapy [102]. Cabozantinib is a relatively new entrant to broad-spectrum TKIs. In the COSMIC-311 trial, it was compared to placebo in previously TKI-treated RAIR-DTC patients, demonstrating superior efficacy with acceptable side effect onset [103].

Vandetanib was tested in RAIR-DTC patients versus placebo in the VERIFY study. Researchers found that this compound failed to obtain an improvement over placebo and in addition introduced an increase in adverse events and deaths [104].

The need for new molecular targets has led to the introduction of sarco/endoplasmic reticulum calcium ATPase (SERCA) inhibitors when RAIR-DTC develops resistance to TKIs. In some studies, SERCAi reached in vitro tumor control after TKI therapy failed [105,106].

In this scenario, the role of single-stranded mature microRNAs (miRNAs) has been investigated. MiRNAs are small sequences of nucleotides that lack coding capability, but are involved in post-transcriptional gene expression. Some miRNAs have been linked to DTC tumorigenesis [107], others have been proposed as biomarker for relapse detection [107,108], and others, such as miR-139-5p, have been suggested as an RAIR pathogenesis explanation [109]. When DTC cells take the way of de-differentiation, this leads to increased aggressivity, metastasis onset, and worse prognosis [2,40,110].

## 8. Conclusions

Understanding DTC functional differentiation requires understanding of its complexity, and it is necessary to build clear criteria for response evaluation. Tumor genomics insights are progressing rapidly, and the chimera of individualized therapy becomes more perceivable real time progresses. Despite that, RAIR-DTC still represents a challenging nosological entity. There are still controversies on RAIR-DTC definition, and post-RAI therapy evaluation with PT-WBS is the only validated criterion of response. Avoiding unnecessary RAI radiation exposure and sub-optimal interventions for patients are current concerns. There is a current need to predict RAIR-DTC before RAI therapy and individualizing therapeutic choices. Thus, molecular imaging is advancing with molecular biochemistry research and should aim for RAIR-DTC prediction, targeted therapy, and optimal onset timing to select second-line treatment strategies in advance.

## Figures and Tables

**Table 1:** ATA 2015 RAIR categories.

I. Malignant/metastatic tissue cannot concentrate RAI on a diagnostic radioiodine scan.
II. Malignant tissue cannot concentrate RAI on a post-^131^I therapy scan.
III. The tumor loses the ability to concentrate RAI after previous evidence of RAI-avid disease.
IV. RAI is concentrated in some lesions only.
V. Metastasis progression even with significant RAI uptake.
VI. >600 mCi of cumulated ^131^I therapy.

Radioactive iodine-refractory (RAIR); radioactive iodine (RAI).
